# Editorial: Assessment of pain in the older population

**DOI:** 10.3389/fpain.2023.1249762

**Published:** 2023-08-25

**Authors:** Pat Schofield, Irmela Gnass

**Affiliations:** ^1^ School of Nursing & Midwifery, Faculty of Health: Medicine, Dentistry and Human Sciences, Paracelsus Medical University, Salzburg, Austria; ^2^ Institute of Nursing Science and Practice, University of Plymouth, Plymouth, United Kingdom

**Keywords:** pain assessment in older adults, pain behaviour, dementia, technology-enabled solution, familiy members

**Editorial on the Research Topic** Assessment of pain in the older population

For many years now we have proposed that pain is regularly assessed and fundamental to the management process. To date, there has been a great deal of research exploring the most appropriate pain assessment tools and great strides have been made in their implementation. Assessment of pain in the older population has presented challenges, especially when there are communication difficulties, as seen in adults with dementia or other communication issues. Pain is not a natural part of the ageing process and people should not be expected to live with it. In recent years, there has been recognition that stoicism does not mean there is no pain and a number of behavioural pain assessment tools have been developed, evaluated, and introduced widely, with widespread implementation of validated pain assessment. The papers in this collection examine the issues of pain assessment in older adults and those with dementia, moving forward thinking on these subjects and presenting innovative ways of implementing pain management using technology. The COVID 19 pandemic resulted in many older adults being isolated at home, so they consequently became more isolated. Furthermore, many services were disbanded in the UK and staff were reallocated to COVID areas which resulted in a reduced number of pain services and therefore increased waiting times.

The first paper in this Research Topic, submitted by the Boston MOBILIZE group, accessed data on 765 older adults living in the community in the Boston area. They used the Brief Pain Inventory to assess the impact of MSK pain on seven domains of psychosocial impact. They found that 67% of their participants reported pain interference in their daily activities. Thus supporting the significant impact on the lives of older adults and opening the door to further work exploring innovative ways of helping older adults manage their pain. Our second paper explores innovative technology as a means of conducting pain assessment in older adults with dementia (PainChek). The study used facial technology to record pain. A sample of 3,144 adults with dementia were recruited through care homes and dementia services in Australia. This large-scale study confirms the hypothesis that facial expression can be a reliable indicator of pain. The next study from the US, explores the role of family caregivers in supporting their relatives with dementia. In a randomised controlled trial that provided training to family members, the authors highlight the significant contribution that can be made in providing personal, emotional, and healthcare needs to individuals with dementia, but they also highlight the considerable strain that this role places on the caregiver. The final study in the collection recruited 45 participants into focus groups, exploring the rapid introduction of technology to implement online pain management during COVID-19. Although small in scale, the study reflected the huge move towards alternative care delivery that took place during the pandemic and the potential successes of these changes.

The studies in this collection focus on key issues relating to pain management in older adults, older adults with dementia, and their family carers. Whilst we have seen great improvements in recent decades in the recognition that pain assessment and pain management are important for all, regardless of age. There is still work to be done and there are areas worthy of further investigation, including innovative methods of assessment, not only of pain but also the impact, caregiver support, and new ways of delivering and supporting care for older adults in the community.

